# Combination of heterologous fibrin sealant and bioengineered human embryonic stem cells to improve regeneration following autogenous sciatic nerve grafting repair

**DOI:** 10.1186/s40409-018-0147-x

**Published:** 2018-04-12

**Authors:** Roghayeh Mozafari, Sergiy Kyrylenko, Mateus Vidigal Castro, Rui Seabra Ferreira, Benedito Barraviera, Alexandre Leite Rodrigues Oliveira

**Affiliations:** 1 Department of Structural and Functional Biology, Institute of Biology, University of Campinas (UNICAMP), Laboratory of Nerve Regeneration, Campinas, SP CEP 13083-970 Brazil; 2 Department of Public Health, Medical Institute of Sumy State University, Sumy, 40007 Ukraine; 3 Center for the Study of Venoms and Venomous Animals (CEVAP), São Paulo State University (UNESP – Univ Estadual Paulista), Botucatu, SP Brazil

**Keywords:** Neurorrhaphy, Sciatic nerve, Human embryonic stem cells, Fibrin sealant, FGF2

## Abstract

**Background:**

Peripheral nerve injury is a worldwide clinical problem, and the preferred surgical method for treating it is the end-to-end neurorrhaphy. When it is not possible due to a large nerve gap, autologous nerve grafting is used. However, these surgical techniques result in nerve regeneration at highly variable degrees. It is thus very important to seek complementary techniques to improve motor and sensory recovery. One promising approach could be cell therapy. Transplantation therapy with human embryonic stem cells (hESCs) is appealing because these cells are pluripotent and can differentiate into specialized cell types and have self-renewal ability. Therefore, the main objective of this study was to find conditions under which functional recovery is improved after sciatic nerve neurorrhaphy. We assumed that hESC, either alone or in combination with heterologous fibrin sealant scaffold, could be used to support regeneration in a mouse model of sciatic nerve injury and repair via autografting with end-to-end neurorrhaphy.

**Methods:**

Five millimeters of the sciatic nerve of C57BL/6 J mice were transected off and rotated 180 degrees to simulate an injury, and then stumps were sutured. Next, we applied heterologous fibrin sealant and/or human embryonic stem cells genetically altered to overexpress fibroblast growth factor 2 (FGF2) at the site of the injury. The study was designed to include six experimental groups comprising neurorrhaphy (N), neurorrhaphy + heterologous fibrin sealant (N + F), neurorrhaphy + heterologous fibrin sealant + doxycycline (N + F + D), neurorrhaphy + heterologous fibrin sealant + wild-type hESC (N + F + W), neurorrhaphy + heterologous fibrin sealant + hESC off (N + F + T), and neurorrhaphy + heterologous fibrin sealant + hESC on via doxycycline (N + F + D + T). We evaluated the recovery rate using Catwalk and von Frey functional recovery tests, as well as immunohistochemistry analysis.

**Results:**

The experiments indicated that sensory function improved when transgenic hESCs were used. The regeneration of sensory fibers indeed led to increased reflexes, upon stimulation of the paw ipsilateral to the lesion, as seen by von-Frey evaluation, which was supported by immunohistochemistry.

**Conclusions:**

Overall, the present data demonstrated that transgenic embryonic stem cells, engineered to overexpress FGF-2 in an inducible fashion, could be employed to support regeneration aiming at the recovery of both motor and sensory functions.

## Background

Following a complete peripheral nerve injury, the primary repair strategy is the so-called “direct nerve repair” or “neurorrhaphy” [1]. Such surgical technique is carried out in two ways: end-to-end (ETE) repair, in which the coaptation is performed between proximal and distal nerve stumps, and end-to-side (ETS) repair, in which the coaptation is performed between the distal nerve stump and another healthy donor nerve [1, 2]. If direct end-to-end repair is not possible, due to lengthy nerve injury and retraction of the stumps, autografting using sensory donor nerves is the gold standard approach.

Thus, whereas suturing the ends of the two nerves together can repair small defects [3], there are cases in which large lesion gaps result from injuries, as well as scars or neuromas, what hinders direct repairing without considerable tension. When the gap is above the critical size, which is about 1 cm in rats, a graft is needed to bridge the damaged ends, reconnecting the proximal and distal stumps [4]. In such situations, ‘autogenous nerve grafting’ is considered as the standard clinical treatment [1, 5]. In this grafting technique, a comparable nerve is first removed from another part of the patient’s body and is used to bridge the gap and connect the two ends of the severed nerve [6, 7]. Without such grafts, these injuries will possibly never heal and can be permanently debilitating [4].

There are several reasons for universal acceptance of autologous grafting in major peripheral nerve repair. The first one is that by taking the donor nerve from within the patient’s body, there is no immune rejection. This procedure offers a cell-rich material through which axons can regenerate and thus has a relatively high success rate in restoring the majority of the functionality to the damaged targets. It offers neuro-supportive architecture (that promotes subsequent regeneration), guidance cues, neurotrophic factors, and a source of Schwann cells [6–8]. Nerve regeneration with autografts usually utilizes much of the graft’s sheath arrangement and topology [4]. By comparison, commercially available substances such as biodegradable polymer and collagen-based hollow tubes have failed to match the regenerative levels of autologous nerve grafting, mainly because they are limited to small defects and show poor functional recovery [9]. Direct nerve repair can be performed using fibrin glue, or nylon suturing; however, the latter is the most common method used for this aim [1].

Although some surgical techniques have proved to lead to better nerve fiber regeneration, the degree of recovery can be highly variable [10, 11]. Therefore, it is crucial to seek for complementary techniques to improve the level of recovery.

In recent years, stem cells have been widely investigated to be used to complement surgery and facilitate the repair of injured peripheral nerves. The sources of these stem cells are widespread and among them are the embryonic stem cells (ESC) routinely derived from the inner cell mass of blastocysts [12–15]. Due to the ability of ESC to self-renew indefinitely and their pluripotency character, they have been considered as an ideal source of cells for biomedical engineering [16].

The effectiveness of ESCs for the treatment of peripheral nerve injury and functional recovery may lie in their ability to differentiate into Schwann cells, secrete neurotrophic factors, promote axon regeneration, and assist in myelin formation (remyelination of axons). Myelination, which determines both regeneration quality and functional recovery, requires the longitudinal wrapping of Schwann cells [12]. In addition, these cells could be induced to express a neural phenotype before transplantation [1].

Advanced cellular bioengineering methods can provide ways to change useful properties of the stem cells according to the objectives of the usage. This can offer opportunities to seek for the treatment of tissues with little or no regenerative capacity including the central nervous system (CNS) and the peripheral nervous system (PNS) [17, 18]. Moreover, the application of growth factors can result in significant increase in nerve regeneration. In this way, fibroblast growth factor 2 (FGF2, also known under the name basic fibroblast growth factor) can offer substantial advantages [19]. The FGF2, a member of the FGF family which comprises 23 members, is encoded by a single copy gene that is alternatively translated to produce one low (18-kDa) and four high (22-, 22.5-, 24-, and 34-kDa) molecular mass isoforms [20, 21]. Recent studies on the function and expression of FGF-2 and its receptors have revealed a physiological role of these molecules in the PNS.

FGF-2 and its receptors are constitutively expressed in the dorsal root ganglia and peripheral nerve [22, 23]. These molecules display an up-regulation in the dorsal root ganglia and the proximal and distal nerve stumps following peripheral nerve injury. In the ganglia, the molecules principally show neuronal expression, whereas, at the lesion site of the nerve, Schwann cells and invading macrophages represent the main cellular sources of FGF-2 and its FGFR1–3 receptors [23]. Whereas Schwann cells are regarded as the main source of FGF-2 [23, 24], the autocrine function of FGF-2 is known to stimulate the Schwann cell proliferation.

Based on the above considerations, the aim of this research was to find conditions under which functional recovery was improved after sciatic nerve neurorrhaphy. We thus used human embryonic stem cells (hESC), genetically modified to overexpress FGF2 in response to the inducer doxycycline, in combination with heterologous fibrin sealant scaffold, to support neuronal survival and regeneration in a mouse model of sciatic nerve injury and repair via autografting with end-to-end neurorrhaphy [25, 26].

## Methods

### Animals and surgical procedures

To investigate the effect of different supplementary compounds (including heterologous fibrin sealant, doxycycline, and hESC) on the site of injury after neurorrhaphy, we designed six groups of eight animals each and followed the procedures described in Table 1.

**Table 1:** Experimental groups and experimental procedures

Tag	Group	Abbreviations	Procedure	Number
1	Neurorrhaphy alone	N	• Immunohistochemistry • Catwalk up to recovery (~ 8 weeks post-surgery) • von Frey	8
2	Neurorrhaphy + heterologous fibrin sealant	N + F	• Immunohistochemistry • Catwalk up to recovery (~ 8 weeks post-surgery)	8
3	Neurorrhaphy + heterologous fibrin sealant + doxycycline	N + F + D	• Immunohistochemistry • Catwalk up to recovery (~ 8 weeks post-surgery) • von Frey	8
4	Neurorrhaphy + heterologous fibrin sealant + wild type hESC	N + F + W	• Immunohistochemistry • Catwalk up to recovery (~ 8 weeks post-surgery) • von Frey	8
5	Neurorrhaphy + heterologous fibrin sealant + hESC off	N + F + T	• Immunohistochemistry • Catwalk up to recovery (~ 8 weeks post-surgery) • von Frey	8
6	Neurorrhaphy + heterologous fibrin sealant + hESC on (doxycycline)	N + F + D + T	• Immunohistochemistry • Catwalk up to recovery (~ 8 weeks post-surgery) • von Frey	8

*N* neurorrhaphy, *F* heterologous fibrin sealant, *D* doxycycline, *W* wild-type hESCs, *T* transgenic hESCs

For sciatic nerve lesion and repair, six to eight-week-old C57BL/6 male mice were obtained from the Multidisciplinary Center for Biological Research (CEMIB), University of Campinas. Both before and after the surgery, the mice were kept in racks with ad libitum access to food and water, under controlled light (light/dark cycle of 12 h) and temperature conditions (i.e., 23 °C). All procedures were done in accordance with the ethical principles regulated by the National Council of Animal Experimentation (CONCEA) and with the approval of the Ethics Committee on Animal Experimentation of University of Campinas (CEUA/UNICAMP, protocol n° 3741–1).

**Fig. 1:**
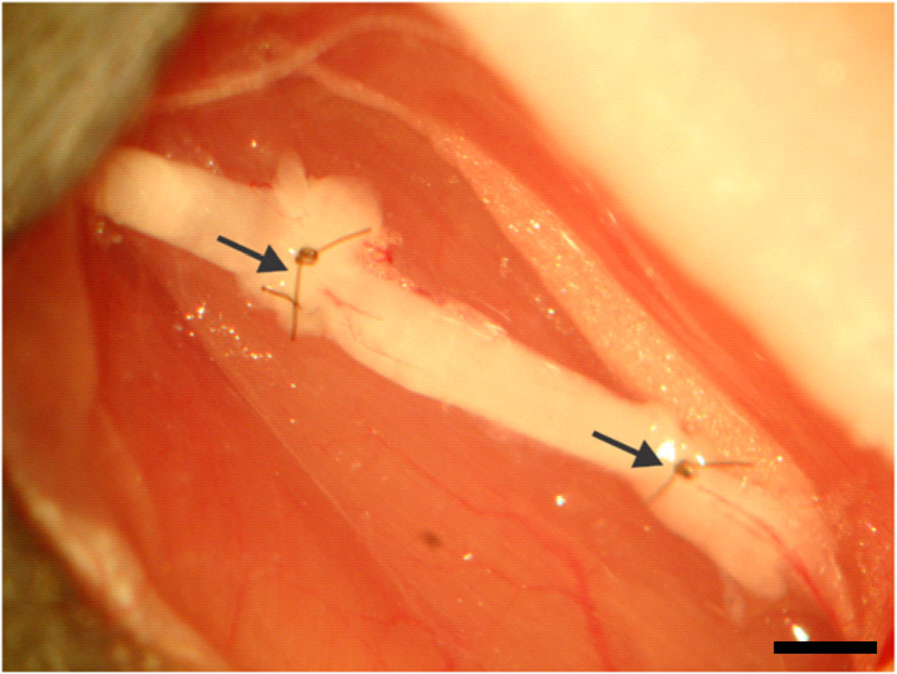
Autografting procedure in which 5 mm of the sciatic nerve of a mouse is transected, rotated 180 degrees, and then is sutured or stitched together by nylon suture and fibrin sealant (20× magnification). Scale bar: 1 mm

The animals were anesthetized with intraperitoneal injections of Kensol (xylazine, Köning, Argentina; 10 mg/kg) and Vetaset® (ketamine, Fort Dodge Animal Health, USA, IA; 50 mg/kg, i.p); totaling 0.12 mL/25 g of the body weight. The left hindlimb of the animals underwent trichotomy. Then around 1.5 cm of the skin was incised with a scalpel. After exposing the sciatic nerve by retracting the musculature, a 5 mm long segment of the nerve was cut out from both ends, rotated 180 degrees, and then inserted between the two nerve stumps. After rotation, the nerve was repaired according to the experimental groups and was sutured under the microscope with 9–0 nylon sutures (Fig. 1). During the surgical procedure, the first two components of the heterologous fibrin sealant were applied, and the third component was added for polymerization. For those groups that embedded the heterologous fibrin sealant (Table 1), the cells were applied to the lesion site (3–5 μL) after adding the third component. The reimplantation stability was tested by gently pulling the nerve or by observing the clots of heterologous fibrin sealant at the site of suture under a microscope.

All the mice underwent surgery and then were maintained in the animal house of the Laboratory of Nerve Regeneration for 60 days, which is the necessary survival time to ensure reinnervation of the target organs. During this period, we implemented a series of motor and sensory evaluations using Catwalk and von Frey tests. After the predetermined survival times, the animals were anesthetized with an overdose of anesthetic (a mixture of xylazine and ketamine). The vascular system was transcardially perfused with 0.1 M phosphate buffer (PB), pH 7.4, and then perfused with 4% formaldehyde in PB (20 mL of fixative per animal). Their sciatic nerve was dissected out and post-fixed in the same fixative solution overnight at 4 °C. They were then cryopreserved in 10, 20, and 30% sucrose buffered solution for 24 h each time, respectively. Before embedding in Tissue-Tek (Miles Inc., USA) and freezing at − 35 °C to − 40 °C, we cut the nerves to yield proximal and distal parts. The longitudinal nerve sections with 12 μm thickness, prepared by a cryostat instrument, were obtained and transferred to gelatin-coated slides and stored at − 20 C until use in immunohistochemistry studies.

All the noted experiments were conducted following the rules of ethics in animal experimentation. We also endeavored to minimize the number of animals and their pain and discomfort.

### Transgenic hESCs

The stems cells used in this research were hESCs derived in the Masaryk University in Brno, Czech Republic [27]. The hESC line CCTL12 was cultured in monolayers on Matrigel as previously described. The hESCs were engineered for inducible overexpression of human FGF-2 as described [18, 28]. Briefly, Tet-On 3G system (Clontech) was used for inducible overexpression. Transfection was done with FuGene HD transfection reagent (Roche, Switzerland). Vectors for stable transfections were used in linearized forms. Selection was done against G-418 at 140 μg/mL and blasticidin at 1.2 μg/mL, according to the pre-determined selection profiles, during two weeks post transfection, in 6-well plates seeded with serially diluted transfected cell suspension. Induction was achieved with 1 μg/mL doxycycline for 24–48 h. The resulting double-stable clone E12–1-1 (overexpressing human FGF-2 in an inducible mode) was used in further experiments. The cell karyotypes were confirmed at Institut für Humangenetik und Anthropologie, Jena, Germany.

### Cell culture

Matrigel (Corning Life Sciences, USA) covered plates were used for culturing hESC in monolayers in conditioned human embryonic stem cell medium (CHESM, see below). After reaching the monolayer, the cells were detached by the TrypLE enzyme (Thermo Fisher Scientific, USA), collected, washed and counted in Neubauer hemocytometer chamber. 300,000 cells were spinned down in 1.5 mL tubes, the medium was aspirated, the cell pellet resuspended in remaining 3–5 μL medium and used in experiments. To prepare the CHESM, we derived mouse embryonic fibroblasts (MEFs) from 12.5-day murine embryos using the standard protocols available in our lab. The MEFs were then frozen and stored at liquid nitrogen for subsequent medium preparations. The fresh HES medium was incubated in the plates with MEF monolayers for 24 h to obtain CHESM.

### hESCs transplantation

**Fig. 2:**
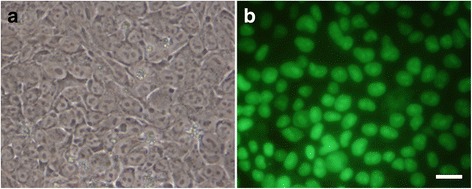
Photographs of hESC activated by doxycycline to overexpress FGF-2. **a** Phase-contrast; (**b**) florescence light. Scale bar: 20 μm

Immediately following neurorrhaphy, 3 × 10^5^ hESCs resuspended in 3–5 μL were engrafted directly at the lesion site together with the heterologous fibrin sealant matrix. To induce overexpression of FGF-2 in the hESCs in vitro, the inducer doxycycline was added to the growth medium at 1 μg/mL for 24–48 h. For experiments in vivo, doxycycline was given to animals combined with the pelleted food as described [29]. Induction was confirmed by GFP expression in the hESCs (Fig. 2).

### Preparation and use of the heterologous fibrin sealant

Heterologous fibrin sealant (F) derived from snake venom was supplied by the Center for the Study of Venoms and Venomous Animals (CEVAP) of São Paulo State University (UNESP), Brazil. Its constituents and instructions for use are provided in the patents n^o^ BR1020140114327 and BR1020140114360. At the time of usage, the components were thawed, mixed, and applied to the sciatic nerves [30–32]. The new scaffold, which is composed of three separate solutions, was homogenized immediately before being used in a final volume of 4.5 μL in the following proportion: fibrinogen (2.5 μL), calcium chloride (1 μL), and thrombin-like fraction (1 μL). During the surgical procedure, the first two components were applied, and the third component was added for polymerization [33–35].

### Immunohistochemistry

In order to visualize the regenerating nerves, we employed an immunohistochemistry technique. This approach enables the detailed observations of nerve regeneration mechanisms in mice.

To accomplish this objective, the slides that were kept in a freezer at − 20 °C, then removed and left at room temperature for a while and, subsequently, washed with 0.1 M PB. Next, the specimens were incubated for 45 min in a 3% BSA solution. The resultant slides were incubated with the primary antibodies reported in Table 2 overnight at 4 °C. After three washes in PB 0.1 M, the respective secondary antibodies conjugated to Cy-3 (1/250, Jackson ImmunoResearch, USA) were applied and incubated for 45 min at room temperature. Finally, the slides were washed and mounted with glycerol/PB (3:1) to obtain immunostained sections.

**Table 2:** Primary antibodies used for immunohistochemistry

Antibody	Supplier	Host	Code	Concentration
Anti-neurofilament	Milipore	Rabbit	Ab1989	1/2000
Anti-ChAT	Millipore	Rabbit	AB143	1/1200
Anti-VGLUT1	Synaptic System	Rabbit	135,303	1/1000
Anti-S100	Dako	Rabbit	Z0311	1/2000

The immunostained sections were observed with a fluorescence microscope (Leica DM5500B, Leica Microsystems CMS GmbH) using the rhodamine filter (CY3). Three representative images were captured from normal and regenerated nerves from different experimental groups using a high-sensitivity camera (Leica DFC345FX, Leica Microsystems CMS GmbH).

For quantification purpose, each immunolabeled picture was segmented into four sub-images to avoid null margins and then measured to obtain the integrated density of pixels using ImageJ software (version 1.33u, National Institutes of Health, USA). For each animal, three individual pictures from different parts of the nerve were collected. In the end, the mean intensity ± standard error was established by averaging the results of segments and pictures for each group. The results also were normalized against the control group (expressed in percentage) and used to compile the bar chart of the experimental groups.

Immunohistochemistry analysis was performed aiming to quantify the following markers:

- Anti-choline acetyltransferase (anti-ChAT) to label motor fibers.
- Anti-neurofilament (anti-NF) to observe regenerated axons or to analyze the organization of the intermediary filaments that comprise the axons of regenerated and contralateral nerves.
- Anti-VGLUT1 to label primary afferent inputs.
- Anti S-100 to characterize the marker of Schwann cells.

### Catwalk test

Following the repair of peripheral nerve injury, the improved behavioral outcome remains the most important evidence for the functionality of axon regeneration. The most frequently used behavioral test to evaluate sciatic nerve injury is the walking tract analysis from the Catwalk XT system (www.noldus.com/animal-behavior-research/products/catwalk).

To perform this test, in a dark room, the animal is placed on a platform with a glass floor (with 100 × 15 × 0.6 cm dimension) fitted with a fluorescent lamp that is used to record the surface trodden by the mouse, and the amount of pressure exerted by its paws, which is directly proportional to the contact area of the floor. Through the glass, the floor of this corridor is monitored by a camera (Pulnix TM-765E CCD) equipped with wide-angle lens. The signal intensity will vary according to the pressure applied by the animals’ paws. The higher the pressure exerted by the paws, the greater the paw contact with the floor and thus the higher the brightness, reflected in the intensity of pixels. These signals are digitized by PC Image-SG frame to frame (Matrix vision GmH, Germany). The catwalk program acquires, stores and analyzes videos of animals roaming the hallway.

The recorded videos were analyzed on a computer by the Catwalk program. For the calculation of motor recovery rate of the sciatic nerve, the amounts related to the distances between the first and the fifth finger (toe spread), and between the third toe and the heel (print length), both the right hind paws (normal) and left (injured) were applied to calculate the sciatic function index (SFI) by the following formula [36]:

$$ \mathrm{SFI}=118.9\ \left[\left(\mathrm{ETS}\hbox{--} \mathrm{NTS}\right)/\mathrm{NTS}\right]\hbox{--} 51.2\ \left[\left(\mathrm{EPL}\hbox{--} \mathrm{NPL}\right)/\mathrm{NPL}\right)\hbox{--} 7.5 $$

Where E is the injured side; N, the normal side; TS, the “toe spread”; and PL, the “print length”. For adaptation and training purposes, all animals are submitted to the test before the sciatic nerve injury.

The Catwalk test and related calculations were performed for all the reported groups in Table 1 in the following way: at the beginning of one-week interval until 14th day followed by four-day intervals until reaching the limit of eight weeks (60 days).

### von-Frey test

Although Catwalk can serve as a standard tool for quantitative and reliable assessment of treatment efficacy, it cannot measure pain, which is an indicator of how good sensory neurons recover [37]. To fill this gap, we considered to include electronic pressure-meter test (von Frey) to our experiments. This test was used to quantify the mechanical sensitivity of the feet following the surgery [38, 39].

To conduct this test, in a quiet room, mice are placed in individual Plexiglas’s box of 12 × 20 × 17 cm in dimension, whose floor consists of a mesh network with a pore size of 5 mm^2^, and a non-malleable 1-mm thickness wire. Mice remain in boxes for 20 min before the experiment to habituate. Mirrors are positioned 25 cm below the testing boxes for easy viewing of the animal paws.

The experimenter is trained to apply – through the mesh network – constant pressure on the plantar surface of the paw until the mouse emits a paw withdrawal reflex, followed by a response characterized as tremor (“flinch”) of the stimulated paw. The stimuli are repeated until the animal shows three similar consecutive measurements (i.e., with a difference of force less than or equal to 10%). When the paw is withdrawn, the instrument automatically records the stimulus force. The maximal force applied was 8 *g*. The hyperalgesia intensity is evaluated by an electronic gauge, which consists of a force transducer connected to a digital counter and is quantified by variation of the nociceptive threshold in grams (gram-force).

We measured the reflexes of the mice from groups 1, 3, 4, 5 and 6 (Table 1) before the surgery to establish a baseline or preoperative sensory function. After performing the surgery, we repeatedly measured the same parameter for eight weeks. Based on the fact that group 2 performed similar to group 1 in the motor behavior, the von-Frey test was not carried out in group 2. Thus, groups 1 and 3 were used as controls.

### Statistical analysis

The results from all the noted experiments are presented as the mean ± standard error of the mean (SEM) and were evaluated by the one-way ANOVA. In all cases, the ANOVA was followed by Bonferroni post-test, assuming a significance level equal to *p < 0.05; **p < 0.01; ***p < 0.001. The resultant data were expressed as the mean ± SEM with *p* < 0.05 being considered to be significant. All statistical analyses were performed using the GraphPad Prism package (GraphPad Software, USA).

## Results

### Expression of FGF-2 by hESCs

We set a demonstration experiment in the lab to ensure that the addition of doxycycline activates the bioengineered cells to overexpress FGF-2. We cultured the cells on a plate, and after reaching the monolayer, doxycycline was given to the medium at 1 μg/mL concentration. The cells were then examined after 24 h under a microscope using phase-contrast filter and florescence light (Fig. 2). The result clearly indicated that the cells have been activated and indeed could fulfill the expected functions.

### Immunohistochemistry

**Fig. 3:**
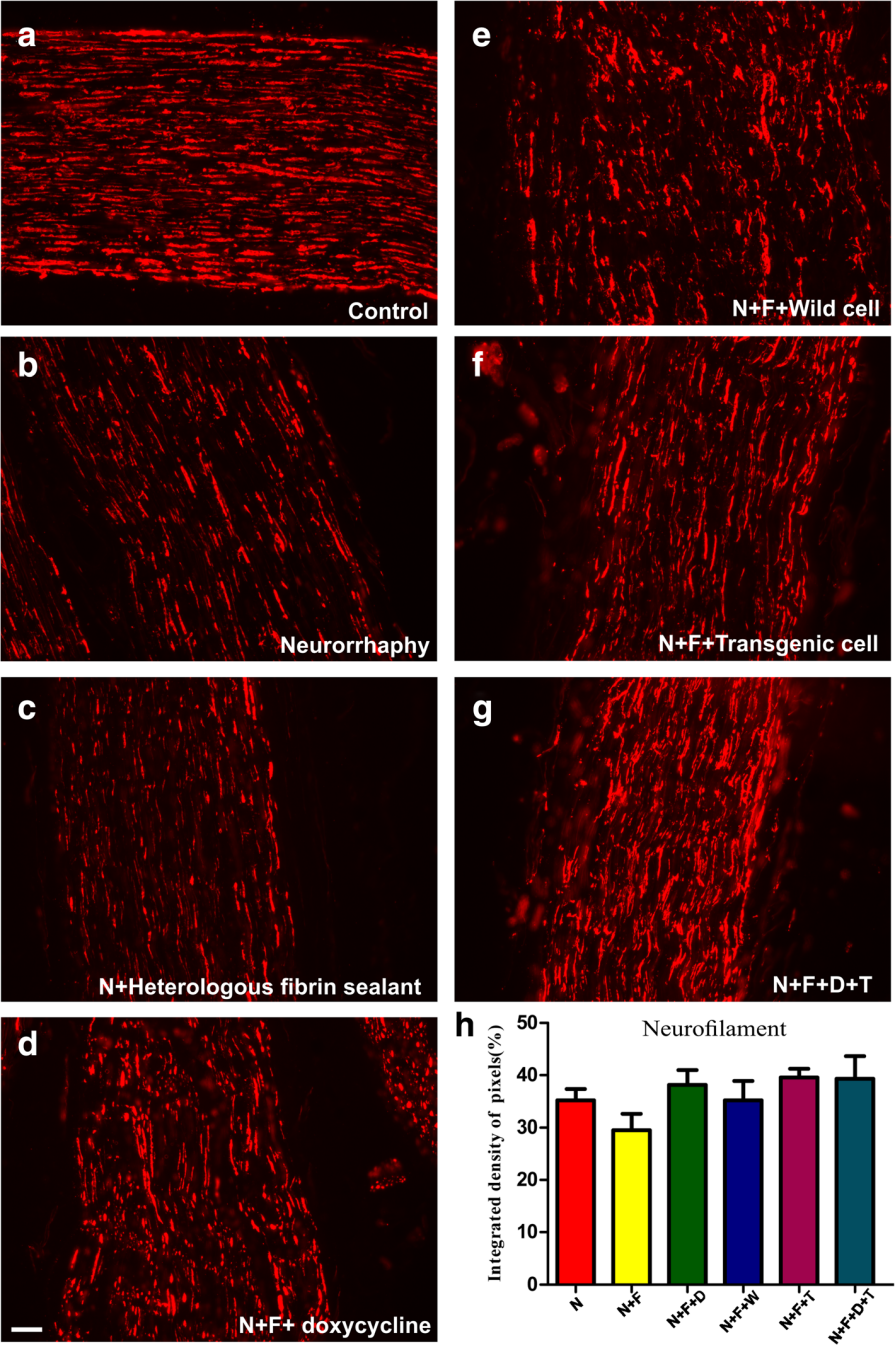
Anti-neurofilament immunostaining of (**a**) the control nerves (**b** to **g**) and all groups, 60 days after surgery. **h** Quantification of the integrated density of pixels in the experimental groups relative to the control group presented in percentage (%) and calculated using Image J software. Statistically, there is no significant difference among the experimental groups. Scale bar: 50 μm. N: neurorrhaphy, F: heterologous fibrin sealant, D: doxycycline, T: transgenic hESCs

Immunolabeling was performed on longitudinal sections of regenerated nerves after 60 days post-injury. By using anti-neurofilament antibody (Fig. 3), we analyzed the organization of the intermediate filaments that constitute the axons of the regenerated and the normal nerves. In all groups, nerve fibers established a parallel pattern along the axis of the nerve, whereas, in the intact (control) nerve, the fibers showed a pattern of parallel waves. Visually, the axons have their highest density in the N + F + D + T group (Fig. 3g) when compared to other groups (~ 40% of the control group) and have the most similar pattern to control group. Nevertheless, statistical analysis performed in relation to the use of this antibody showed no significant difference among the experimental groups (Fig. 3h). The mean intensities of the immunostainings quantified by integrated pixel density are displayed in Table 3.

**Table 3:** Quantification of the immunostaining by the integrated pixel density – ratio ipsi/contralateral (%)

Antibody	Anti-VGLUT1	Anti-S100	Anti-neurofilament	Anti-ChAT
N (norm)	66.19 ± 7.752	150.6 ± 15.67	35.18 ± 2.189	47.66 ± 5.526
N + F	57.04 ± 9.484	100.5 ± 9.04	29.51 ± 3.091	50.26 ± 7.681
N + F + D	54.36 ± 6.536	80.04 ± 6.90	38.15 ± 2.819	48.95 ± 5.090
N + F + W	73.84 ± 6.312	96.24 ± 4.91	35.18 ± 3.739	53.51 ± 7.145
N + F + T	81.91 ± 10.41	131.5 ± 16.37	39.56 ± 1.682	56.79 ± 6.056
N + F + D + T	107.3 ± 15.88	99.7 ± 12.42	39.30 ± 4.336	61.13 ± 5.792

*N* neurorrhaphy, *F* heterologous fibrin sealant, *D* doxycycline, *W* wild-type hESCs, *T* transgenic hESCs

**Fig. 4:**
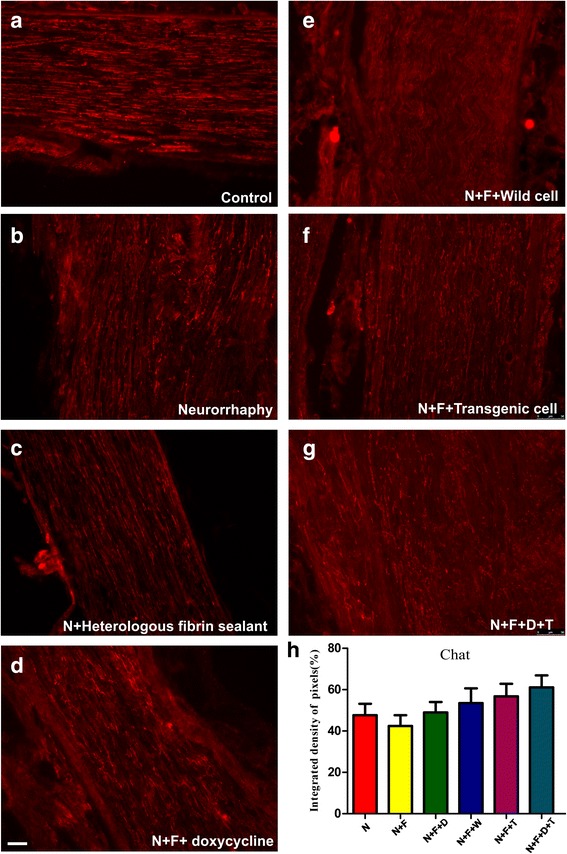
Anti-ChAT immunostaining of (**a**) the control nerves and (**b** to **g**) all groups, 60 days after surgery. **h** Quantification of the integrated density of pixels in the experimental groups in relation to control group (%). Statistically, there is no significant difference among experimental groups. Scale bar: 50 μm. N: neurorrhaphy, F: heterologous fibrin sealant, D: doxycycline, T: transgenic hESCs

The choline acetyltransferase (ChAT) – the enzyme responsible for the biosynthesis of acetylcholine – is used currently as the most specific indicator for monitoring the functional state of cholinergic neurons in the peripheral nervous systems. The anti-ChAT showed intense motor axons in the control group (Fig. 4); albeit for the experimental groups, the motor axons are less intense. The mean intensities of the immunostaining quantified by integrated pixel density are displayed in Table 3. Despite the incremental trend from N + F (~ 43%) towards N + F + D + T group (~ 60%), statistical analysis performed for this antibody showed no significant differences between the groups.

**Fig. 5:**
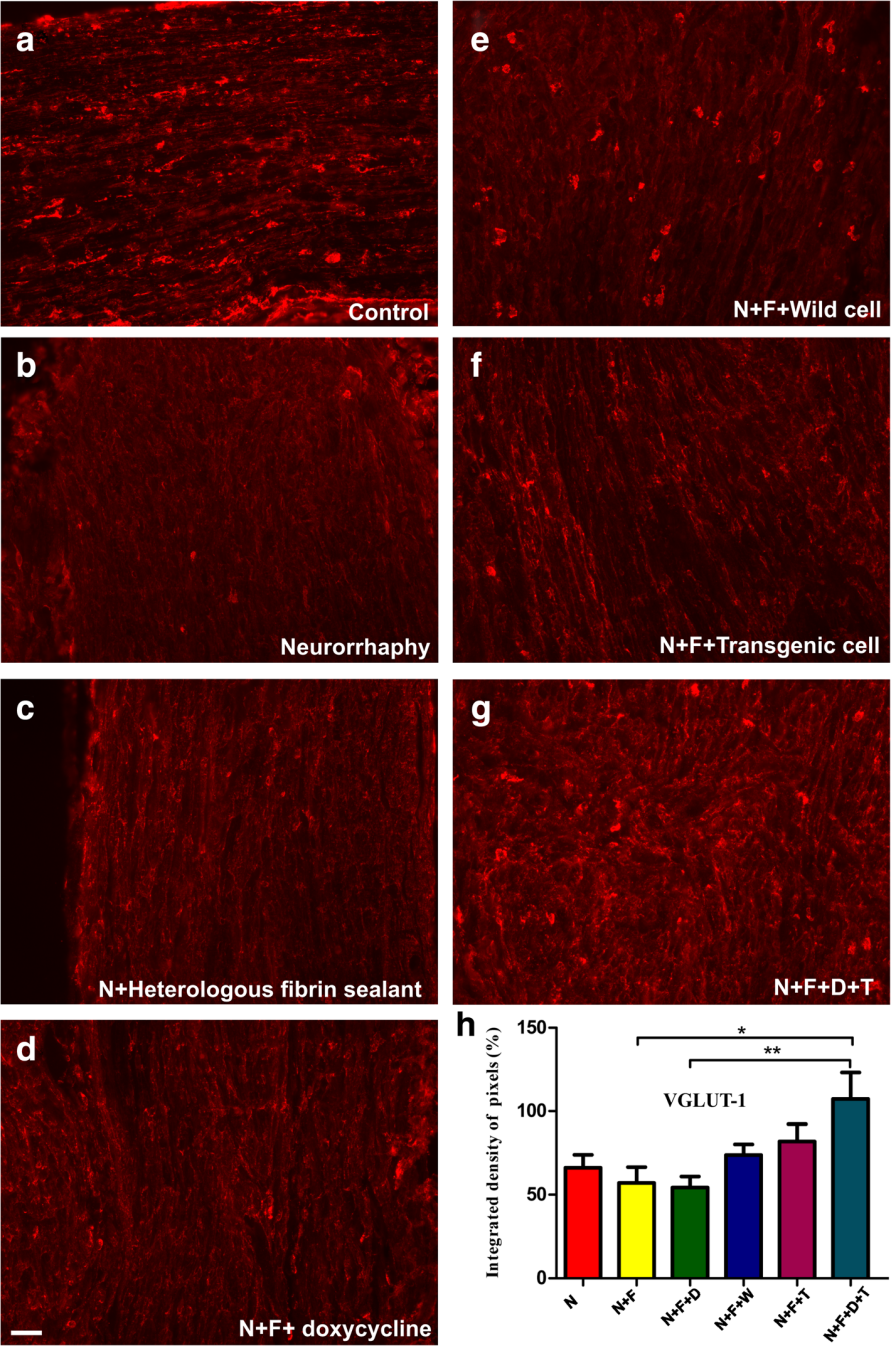
Anti-VGLUT1 immuno-staining of (**a**) the control nerves and (**b** to **g**) all groups, 60 days after surgery. **h** Quantification of the integrated density of pixels in the experimental groups relative to control group (%). Statistically, the difference between N + F versus N + F + D + T and N + F + D versus N + F + D + T groups are meaningful with *p* < 0.05 and *p* < 0.01, respectively. Scale bar: 50 μm. N: neurorrhaphy, F: heterologous fibrin sealant, D: doxycycline, T: transgenic hESCs

Using anti-VGLUT1 antibody (Fig. 5), which is a marker of sensory neurons, we labeled the primary afferent inputs responsible for transport of glutamate into the synaptic vesicle. The observation with a fluorescence microscope indicated that VGLUT1 antibody is associated with a more sensory neuron in the N + F + D + T group (Fig. 5g). The mean intensities of the immunostainings are reported in Table 3. Statistical analysis performed for this antibody showed statistically significant differences between the experimental groups with the N + F + D + T one having the highest integrated density. Based on this analysis, the N + F + D + T group is yielding the same level of sensory neurons as the control group (Fig. 5h).

**Fig. 6:**
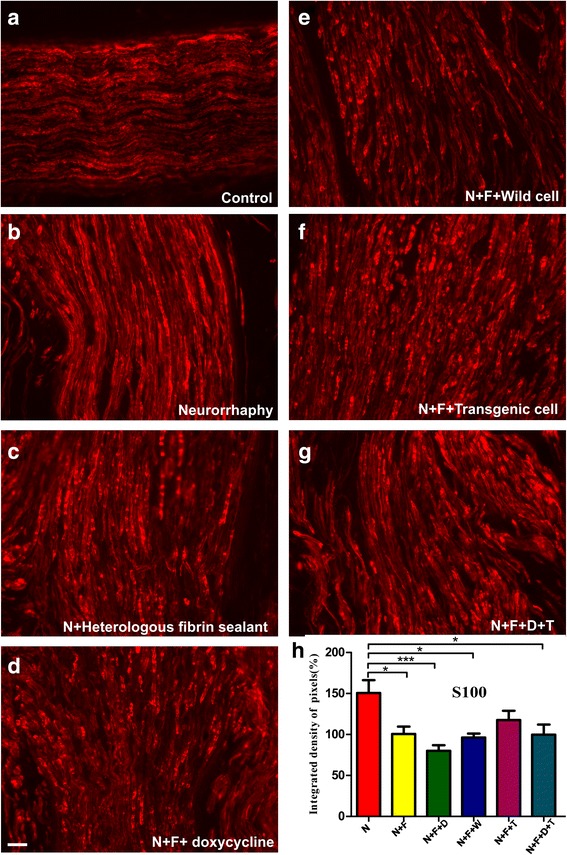
Anti-S100 immuno-staining of (**a**) the control nerves and (**b** to **g**) all groups, 60 days after surgery. **h** Quantification of the integrated density of pixels in the experimental groups relative to control group (%). Statistically, the difference between the following groups are meaningful: N versus N + F (*p* < 0.05), N versus N + F + D (*p* < 0.001), N versus N + F + W (*p* < 0.05), and N versus N + F + D + T (*p* < 0.05). The N versus N + F + T shows no significant difference. Scale bar: 50 μm. N: neurorrhaphy, F: heterologous fibrin sealant, D: doxycycline, T: transgenic hESCs

The anti-S100 staining (Fig. 6), a characteristic marker of Schwann cells, was intense on N and N + F + T groups (150% and 120%, respectively), but in the group that incorporated transgenic cells was at the same level as the control group (100%). The mean intensities of the immunolabels, quantified through the integrated density of pixels are reported in Table 3. Similar to the VGLUT1 antibody, the statistical analysis shows a meaningful difference between the experimental groups.

### Motor evaluation of functional recovery via Catwalk

**Fig. 7:**
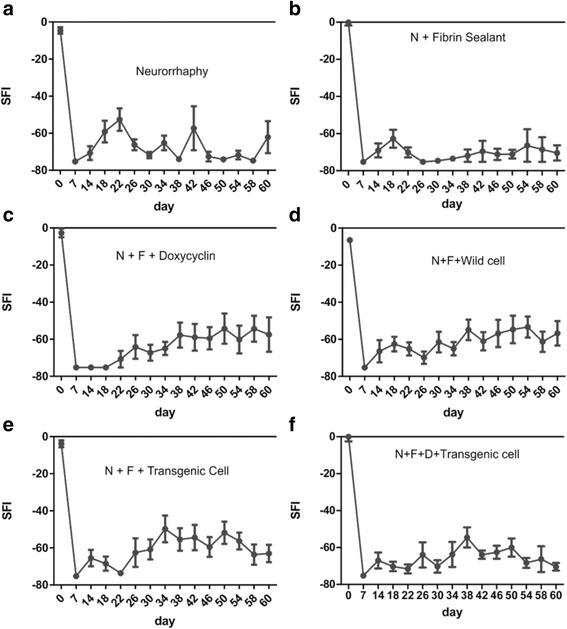
The results achieved from Catwalk test separated by groups and calculated using the SFI index. **a** Neurorrhaphy group. **b** Neurorrhaphy + heterologous fibrin sealant. **c** Neurorrhaphy + heterologous fibrin sealant + doxycycline. **d** Neurorrhaphy + heterologous fibrin sealant + wild type hESCs. **e** Neurorrhaphy + heterologous fibrin sealant + transgenic cells (non-induced). **f** Neurorrhaphy + heterologous fibrin sealant + doxycycline + transgenic cells induced

The detailed results of Catwalk test separated by days and groups are summarized in Fig. 7. In all groups, in the first measurement session (seven days after the surgery), the SFI was in the lowest level of − 75, meaning that the mice could not use their paws at all. However, after the second week, the changes started. This was marked by a gradual increase in the SFI values in most of the groups. In the case of N + F group, the gradual increase in using the injured paw, which was indicated by a higher SFI value, dropped after 4th session (day 22) and remained constant for the remaining duration of the experiment, whereas in the N + F + D group, this trend started 22 days after and gradually increased afterwards. A similar trend could be seen in the N + F + W group. In contrast, the incremental trend in N + F + T and N + F + D + T groups reversed after around 34–38 days.

In the pre-operation measurements, there were slight variations (5 ± 2.5) in the SFI score of all groups that seemingly were due to the intrinsic error of the instrument/technique and also the personal walking habit of mice. Although the improvement of injured paws started after around two weeks (14–18 days), the most noticeable changes began after a month (between 34-38th days). However, the improvement was not sustainable and fluctuated throughout the study period. The improvement was also highly variable among groups. The best scores belonged to those groups that incorporated hESCs, whereas the group with no additive (N) compounds or a simple one like heterologous fibrin sealant (N + F) showed no recovery at all.

The *p*-value for Catwalk experiments was calculated based on groups and days. When the one-way statistical test was performed for the results classified according to days, except for the 22th and 34th days in which their *p*-values were significant (N versus N + F, N versus N + F + D, and N versus N + F + D + T with *p* < 0.05 and N versus N + F + T with *p* < 0.01 for day 22nd and N + F versus N + F + T with *p* < 0.05 for day 34th), the groups in other days showed no significant differences. Similarly, when the t-test was performed for the results divided into groups, only the N + F versus N + F + D (*p* < 0.05), N + F versus N + F + W (*p* < 0.01), and N + F versus N + F + T (*p* < 0.01) showed significant differences. The general trend observed in Catwalk results, however, is the absence of significant statistical differences among the experimental groups.

### Sensory function evaluation via Von Frey test

**Fig. 8:**
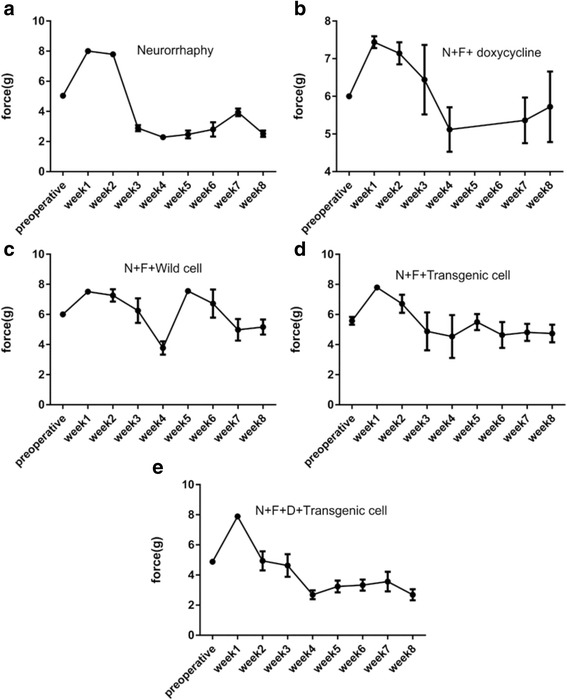
The results of von Frey test for the following groups during eight weeks period. **a** Neurorrhaphy group. **b** Neurorrhaphy + heterologous fibrin sealant + doxycycline. **c** Neurorrhaphy + heterologous fibrin sealant + wild type hESCs. **d** Neurorrhaphy + heterologous fibrin sealant + transgenic cells (non-induced). **e** Neurorrhaphy + heterologous fibrin sealant + doxycycline + transgenic cells (induced)

The results obtained from von Frey test are shown in the Fig. 8. The pre-operative results for healthy mice commonly vary between 5 and 6 *g* (force), which is the case for all the groups. Following sciatic nerve injury (first week) that leads to the loss of sense of the paw, the stimulus force reaches a maximum of around 8 g. After this peak, the trend reverses, and the required stimulus force decreases in several successive weeks until reaching a minimum in week 4. In groups in which transgenic cells were incorporated, the curves almost flatten after this minimum and show little change until the end of the period.

## Discussion

Peripheral nerve injury is a worldwide clinical problem that impairs the quality of life of the patient. Thus, the recovery from a severe nerve transection is usually far more difficult, and the results are less satisfactory [40]. To improve the degree of nerve regeneration and functional recovery, cell transplantation therapy has been used with some degree of success. In contrast to other varieties of stem cells, hESCs can self-renew indefinitely and differentiate into a diverse range of specialized cell types making them an important source for transplantation therapy and biomedical engineering [16, 40]. FGF-2, as a constituent of hESC growth medium, is the most significant regulator of the hESC self-renewal. In practice, since it is difficult to deliver the active growth factors over the entire duration of regeneration in a controlled manner, genetically modified cells are used to deliver a continuous supply of active growth factors. The gene expression in these modified cells can be turned on by using doxycycline as the regulator for inducible gene expression systems.

Immunolabeling results obtained 60 days after cell therapy were comparable to those already reported in the literature. The study of nerves by anti-neurofilament antibodies demonstrated that the axons regenerated to some extent in all experimental groups. The finest nerve regrowth and the most uniform pattern of axons was observed in the N + F + D + T group. The fact that all groups showed axon regeneration to some extent (around 40% of the control group) alongside with the absence of inter-group statistical differences imply that ‘autografting’ has been successful in stimulating fiber nerve regeneration. However, this antibody cannot indicate the type and accuracy of nerve sprouting. To distinguish between them, ChAT and VGLUT-1 antibodies were employed to label motor and sensory axons, respectively.

Based on anti-ChAT results, the lowest number of motor axons were observed in the N + F group meaning that heterologous fibrin sealant alone added to the site of injury offers no benefits to nerve regeneration. Although the N + F + D + T group does not stand out as a group with the best motor neuron regeneration, it possesses the highest recorded motor neurons labeling (~ 60% compared to control group). In line with the present results, it is known that the number of regenerated motor neurons after sciatic nerve transection is not optimal [41]. In our experiments, statistical analysis revealed no statistical differences between experimental groups as well as between the N + F + D + T group and control group. This may suggest that cell therapy (hESC inducibly overexpressed FGF-2) alone is not quite sufficient to facilitate motoneuron recovery and there is a need for supplementary approaches, such as the use of neurotrophic factors to promote motoneuron regeneration.

The addition of cells provided the most prominent results regarding sensory neuron reinnervation. For instance, wild type and transgenic cells have increased the percentage of sensory axons labeling respectively by 73 and 82% relative to control group (for comparison, this value for the neurorrhaphy-alone group was 66%). When the transgenic cells were activated by doxycycline, the intensity rate increased to the same level as a control group (~ 104%). This significant increase in nerve regeneration is believed to be linked to the expression of FGF-2 growth factor because when doxycycline alone was added to the site of injury (N + F + D group), it was not very effective in stimulating the regeneration. In the peripheral nervous system, FGF-2 modulates neuronal survival, prevents the lesion-induced death of sensory neurons, and stimulates nerve regeneration [42]. The group N + F + D + T, when analyzed by S100 immunolabelling showed less activated Schwann cell and improved axon regeneration. In fact, the labeling of Schwann cells was identical to control group (~~100%) meaning that the combined effect of transgenic cells and heterologous fibrin sealant has been successful in supporting Schwann cells at the injury site. This also suggests that the remyelination process was stimulated by the transplanted cells [12].

As was marked by S100 antibody, except for the neurorrhaphy group, all other groups that incorporated heterologous fibrin sealant glue showed normal intensity of Schwann cells and thus a more stable endoneurial microenvironment. Whereas Pabari et al. [43] have demonstrated the constructive role of fibrin sealants in producing less inflammation, less fibrosis, better axonal regeneration, and better fiber alignment, based on our immunohistochemistry results, we can only confirm its crucial role in reducing inflammation and establishing a framework capable of retaining the grafted cells. This is corroborated by the absence of statistical differences between the neurorrhaphy-alone group and others in the outcomes of neurofilament immunolabeling.

In the context of behavioral patterns, in the first week after the surgery, as expected, none of the mice were able to use their paws, and thus the SFI values obtained from Catwalk test were equal to − 75. This is due to the total loss of function of the nerve after neurorrhaphy and indeed a clue for successful lesion/repair process [44]. In most of the groups, it took about a month (26–30 days) for the mice to be able to show recovery signs. In some instances, mice were able to use their paws, however, due to likely inflammation and relevant pain, SFI remained at − 75. The inflammation/hyperalgesia hypothesis is further supported by the results of von-Frey test [39]. Coincidently, at the 4th week, the force to stimulate the nerve reflex was reduced to a minimum of about 4 *g*. It is important to highlight that, in fully denervated muscle, the highest level of post-synaptic sprouts occurs four weeks after injury and repair [41]. This could further explain the hypersensitivity of mice to pain at this specific time.

Despite the slight initial improvement, group N + F showed no recovery in the nerve function. The SFI score from this group remained close to − 75 after 26 days in almost all subjects. The fact that the results of this group showed no advantages in relation to the neurorrhaphy-alone group implies that the inclusion of heterologous fibrin sealant in the site of injury has no recovery effects. This finding indeed is in agreement with the results of immunohistochemistry methods discussed above. However, it is important to emphasize that the new heterologous fibrin sealant from CEVAP has the property of working as a scaffold that secures stem cells at the site of injury. In turn, its use is of great value for cell therapy approaches [28, 32, 45–49].

Among the studied groups, those that included doxycycline, wild hESC, and transgenic cells achieved better results. All these groups showed recovery to some extent from the first month onward, though the results were not stable and varied among measurement sessions. The reversal of SFI in the N + F + T and N + F + D + T groups, however, is linked to more painful paws arising from higher sensory nerve regeneration as was demonstrated by immunohistochemistry studies and von-Frey test. The neuropathic pain is known to develop after sciatic nerve injury. Hence, differences in SFI levels may not only reflect motor-related impairments but also be pain-related due to decreased weight load on the affected paw [50, 51]. As was stated by Deumens et al. [50], “functional impairments may be purely related to pain behavior, and the behavioral effects may be a compromise between pain-related and motor-related alterations”. Following autografting, the mice started to use their heel to contact the ground rather than their toes. The origin of this behavior is believed to be motor-related, rather than pain related, for it began within days after injury, whereas pain sensitivity, as indicated by von-Frey test, was only observed after a delay of about 3–4 weeks. Recovery of pain sensitivity was also corroborated by toe pinch reflex test on awake mice. The animals displayed a blink reflex or a withdrawal response to light paw-pinching.

To the best of our knowledge, this is the first time that sciatic nerve regeneration has been evaluated by combined behavioral tests, namely von-Frey and Catwalk, in a mouse model. The former can provide information on sensory nerve regeneration whereas the latter (or its substitute; the walking track analysis) gives information about motor neuron regeneration and their aggregation translated into functional recovery.

Functional recovery after complete nerve injury depends on several factors including regrowth of axons, specific reinnervation of the target region, and maturation of nerve fibers and reinnervated muscle fibers [52, 53]. Reinnervation of muscle fibers includes the establishment of neuromuscular connections and formation of motor units as an essential element of force development and movement control. In this phase, nerve fibers should grow from the proximal nerve stump and reconnect to muscle fibers. Naturally, nerve fibers incorporate a small number of muscle fibers, however, during the nerve regeneration, the muscle fiber group within the same motor unit and the correspondent regenerated axons incorporate additional muscle fibers. These fibers that bear the same biochemical signatures compensate for reduced number of axons that succeed in reaching the denervated muscle [41]. The fact that motor axons reinnervate (enlarge) more than the normal number of muscle fibers causes all denervated muscle fibers to be innervated by as few as 20–25% of the normal number of motor axons [41]. As a consequence, poor functional recovery is recorded by catwalk experiment. In other words, the absence of functional recovery does not necessarily mean failure in nerve regeneration. Instead, “the misdirection of regenerating axons (inaccurate reinnervation) could strongly affect sensory-motor skills, which require accurate reinnervation of the proper muscles and receptors, whereas gross functional responses are more dependent on the amount of reinnervation rather than its accuracy” [54].

## Conclusions

The application of human embryonic stem cells (hESCs) modified to inducibly overexpress FGF-2 to the site of injury was successful in the regeneration of sensory and motor fibers as demonstrated by von-Frey test and immunohistochemistry analysis.

Based on the present study, the new heterologous fibrin sealant from CEVAP can facilitate nerve repair, which corroborates previous publications in the field [55–58]. We also believe that for higher functional recovery and better motor neuron reinnervation, fibrin sealant and cell therapy should be used in combination with neurotrophic factors.

## Abbreviations

CEMIB: Multidisciplinary Center for Biological Research
CEUA: Ethics Committee on Animal Experimentation of University of Campinas
CEVAP: Center for the Study of Venoms and Venomous Animals (Brazil)
ChAT: choline acetyltransferase
CHESM: conditioned human embryonic stem cell medium
CNS: central nervous system
D: doxycycline
DMEM: Dulbecco’s modified Eagle’s medium
ESC: embryonic stem cells
ETE: end-to-end neurorrhaphy
ETS: end-to-side neurorrhaphy
F: heterologous fibrin sealant
FGF2: fibroblast growth factor 2
hESC: human embryonic stem cells
MEFs: mouse embryonic fibroblasts
NF: neurofilament
NGF: nerve growth factor
PNS: peripheral nervous system
SEM: standard error of the mean
SFI: sciatic function index
VGLUT1: vesicular glutamate transporter type 1
